# Independent and Combined Effects of Socioeconomic Status (SES) and Bilingualism on Children’s Vocabulary and Verbal Short-Term Memory

**DOI:** 10.3389/fpsyg.2017.01442

**Published:** 2017-08-25

**Authors:** Natalia Meir, Sharon Armon-Lotem

**Affiliations:** ^1^Department of English Literature and Linguistics, Bar Ilan University Ramat Gan, Israel; ^2^Gonda Multidiciplinary Brain Research Center, Bar Ilan University Ramat Gan, Israel

**Keywords:** child bilingualism, verbal short-term memory, socioeconomic factors, Russian–Hebrew, lexicon, sentence repetition

## Abstract

The current study explores the influence of socioeconomic status (SES) and bilingualism on the linguistic skills and verbal short-term memory of preschool children. In previous studies comparing children of low and mid-high SES, the terms “a child with low-SES” and “a child speaking a minority language” are often interchangeable, not enabling differentiated evaluation of these two variables. The present study controls for this confluence by testing children born and residing in the same country and attending the same kindergartens, with all bilingual children speaking the same heritage language (HL-Russian). A total of 120 children (88 bilingual children: 44 with low SES; and 32 monolingual children: 16 with low SES) with typical language development, aged 5; 7–6; 7, were tested in the societal language (SL-Hebrew) on expressive vocabulary and three repetition tasks [forward digit span (FWD), nonword repetition (NWR), and sentence repetition (SRep)], which tap into verbal short-term memory. The results indicated that SES and bilingualism impact different child abilities. Bilingualism is associated with decreased vocabulary size and lower performance on verbal short-term memory tasks with higher linguistic load in the SL-Hebrew. The negative effect of bilingualism on verbal short-term memory disappears once vocabulary is accounted for. SES influences not only linguistic performance, but also verbal short-term memory with lowest linguistic load. The negative effect of SES cannot be solely attributed to lower vocabulary scores, suggesting that an unprivileged background has a negative impact on children’s cognitive development beyond a linguistic disadvantage. The results have important clinical implications and call for more research exploring the varied impact of language and life experience on children’s linguistic and cognitive skills.

## Introduction

Socioeconomic status (SES) and bilingualism have been found to impact the development of preschool children, yielding variation in their linguistic and cognitive profiles. Previous studies consistently demonstrate effects of SES on language development (e.g., Locke et al., 2002; Hart and Risley, 2003; Qi et al., 2006). Performance of children from low SES groups is reported to be three-quarters to one standard deviation below scores for the general population (for an overview see Roy and Chiat, 2013). Similarly, previous research demonstrates that sequential bilingual children with typical language development perform significantly lower than their monolingual peers on standardized language tests, which are normed on monolingual children (e.g., Restrepo, 1998; Bedore and Peña, 2008). Low language performance of children from low SES backgrounds and bilingual children leads to disproportionately high rates of identification of Specific Language Impairment (SLI) among these groups (for more detail see Roy and Chiat, 2013; Armon-Lotem and de Jong, 2015). The results of the current study are expected to deepen our understanding of how environmental factors (SES and bilingualism) affect cognitive and language skills in preschool children and help educators and speech and language pathologists tease apart disorder and variation due to environmental impacts. The current study addresses this problem by exploring the influence of SES and bilingualism on expressive vocabulary and verbal short-term memory of preschool children. In previous studies comparing children of low and mid-high SES, the terms “a child with low-SES” and “a child speaking a minority language” are often used interchangeably, precluding a differentiated evaluation of these two variables.

Moreover, in the few studies that attempted to address the differentiated impact, bilingual children came from widely mixed language, ethnic and cultural groups (Calvo and Bialystok, 2014; Chiat and Polišenská, 2016). In the present study, all bilingual children were born in Israel, attended the same kindergartens as the monolingual children and spoke the same heritage language (HL-Russian). This allowed us to focus on independent and combined effects of SES and bilingualism on expressive vocabulary and three repetition tasks among children who are monolingual and bilingual speakers of Hebrew, the societal language (SL). Repetition tasks [nonword repetition (NWR) and sentence repetition (SRep)] are reliable screening measures for diagnosing SLI among monolingual and bilingual children (e.g., Conti-Ramsden et al., 2001; Armon-Lotem and Meir, 2016). Yet research on effects of SES on repetition tasks is scarce (but see Balladares et al., 2016; Chiat and Polišenská, 2016). Likewise, few studies have evaluated separate and combined effects of SES and bilingualism on repetition tasks (but see Chiat and Polišenská, 2016).

Repetition tasks [including forward digit span (FWD), NWR, and SRep] tap into verbal short-term memory, but also activate long-term memory representations (for an overview see Meir, 2017). Baddeley (2001) suggests that verbal short-term memory storage is facilitated by lexical-semantic and morpho-syntactic knowledge stored in long-term memory. Repetition tasks differ in their linguistic load. For example, FWD carries the lowest linguistic load and is generally viewed as a pure cognitive measure of verbal short-term capacity (Richardson, 2007). This is supported by weaker correlations between FWD and vocabulary, as compared to correlations between NWR and vocabulary size (Baddeley et al., 1998). Contrastingly, SRep draws more on long-term memory representations than on verbal short-term memory, as the task draws on phonological, lexical, morphological, syntactic, and semantic information stored in long-term memory. Thus, the evaluation of independent and combined effects of SES and bilingualism is expected to deepen our understanding on how environmental factors shape children’s language and cognitive skills.

The introduction is structured as follows: an overview of studies evaluating the influence of SES on language development of monolingual children with emphasis on repetition tasks is followed by a brief overview of previous findings on the effects of bilingualism on tasks tapping into verbal short-term memory. The introduction will conclude with a presentation of what is currently known of the combined impact of bilingualism and SES, outlining the research questions addressed in the present study.

### Effects of SES in Monolingual Children

Children from disadvantaged backgrounds exhibit poorer linguistic skills as measured by standardized tests based on developmental norms (e.g., Qi et al., 2006). Due to impoverished language input, children from low SES often perform in the SLI range when assessed on language screening tests: the gap between children from low and high SES is observed on all standardized measures of English (Roy and Chiat, 2013). There is also a strong association between child vocabulary size and SES: children from mid-high SES families have bigger vocabulary sizes than their peers from disadvantaged homes (e.g., Dunn and Dunn, 1981; Hoff, 2003, 2006; Pan et al., 2005). Strikingly, the gap between low and mid-high SES in vocabulary and language processing skills is already evident as early as the age of 18 months, and by age of 24 months this gap presents a 6-month disadvantage (Fernald et al., 2013). Moreover, children from mid-high SES homes develop better morpho-syntactic abilities. In Hebrew, Schiff and Ravid (2012) showed that monolingual Hebrew-speaking children from low SES (as measured by their neighborhood) were consistently less accurate than their peers from high SES families on nominal and adjectival formation across all school grades (Grades 1–5). In a more recent study by Levie et al. (2017), monolingual Hebrew-speaking children with and without SLI from mid-high and low SES aged 6–14 were compared on derivational morphology. The findings showed that typically developing children from the mid-high SES group obtained the highest scores, and the language impaired low SES group always scored lowest. Interestingly, typically developing children from the low SES group often showed similar performance to that of language impaired children from mid-high SES groups. The results indicate that the effects of SES make it difficult to disentangle disadvantaged background and disorder.

Regarding repetition tasks, previous findings are inconsistent. Some studies show a negative effect of low SES on children’s performance on repetition tasks, while others show that there is no difference between children from low and mid-high SES. For example, for FWD as a cognitive measure of verbal short-term memory capacity, previous research reported cultural biases for children aged 5–12 (Jensen and Figueroa, 1975). Similarly, for repetition tasks with a larger linguistic load (NWR and SRep), Gardner et al. (2006), studying a group of 668 British English-speaking children, reported effects of geographical location, but not parental occupation. Geographical location and parental occupational status are often used as indices of SES.

Conversely, the negative effect of low SES on verbal short-term memory tasks was not observed in a study by Engel et al. (2008), who investigated the effect of SES on expressive and receptive vocabulary and verbal short-term memory in 40 Brazilian children aged 6–7. The SES index was comprised of three measures (monthly family income, occupational status, and education of a main caregiver). The authors found a negative effect of SES on expressive and receptive vocabulary: children from low SES families scored significantly lower as compared to their peers from high SES families. The authors found neither effect of SES on cognitive measures (e.g., FWD) nor on NWR, a measure of verbal short-term memory more linked to vocabulary. However, the authors suggest that their results should be interpreted with caution. The effect size for NWR was small, suggesting that, indeed, NWR is a measure independent of SES influence. The effect size, however, for FWD was moderate (as measured by Cohen’s *d*) indicating that with a larger sample size the effect of SES on FWD might reach significance.

The effect of SES on sentence memory was evaluated by Alloway et al. (2014) in British children aged 4; 3–5; 8. A negative effect of low SES was observed for the SRep task. Similar to other studies conducted in the United Kingdom, SES status was determined by a classification of residential neighborhoods. The authors concluded that long-term memory representations were affected by SES as reflected in lower scores on the SRep task, which taps into syntactic and semantic knowledge. However, the authors showed that repetition tasks of lower linguistic load (e.g., repetition of real words) were not influenced by SES.

The effect of SES on NWR and SRep was further addressed in a large-scale study of British English-speaking children by Roy et al. (2014). A total of 208 children from low SES families and 168 children from mid-high SES families, aged 3; 6–4; 11, were compared on receptive and expressive vocabulary and on NWR and SRep tasks. The children were split into SES groups based on the Index of Multiple Deprivation computed for the geographical area in which they reside. The sample comprised of mainly children for whom English was their only language; however, children with an additional language at home were not excluded from the study. The authors reported that the distribution of children with an additional language at home did not differ across the two SES groups. The results indicated that low SES children scored significantly lower on receptive and expressive vocabulary, demonstrating a negative effect of low SES. Furthermore, the findings indicated significant group differences on NWR and SRep tasks, with children from low SES showing significantly lower scores. The authors showed that proportions of low scorers on the NWR and SRep tasks were eight times greater in the low SES group as compared to the mid-high SES group. Strong associations were observed between children’s performance on vocabulary and repetition tasks. The results further indicated that the gap between low and mid-high SES groups narrows with age. The only measure which showed no differences between children from low and mid-high SES was the function-word score on the SRep task: children from low SES backgrounds performed in the expected range for this measure.

A recent study by Balladares et al. (2016) investigated effects of SES on NWR and SRep tasks in 126 typically developing Spanish-monolingual Chilean children, aged 3; 10–6; 3, from low and high SES. The SES status was obtained based on the type of school that children were attending: low-SES participants were recruited from public schools and high SES participants were drawn from private schools. The segregation between public and private schools is reported to also be related to family income. Similar to Roy et al. (2014), there was an effect of SES on SRep; however, as observed in Engel et al. (2008), NWR was found to be free of an SES effect. A negative effect of SES was also observed for receptive vocabulary, and when this was controlled for, the effect of SES on SRep disappeared.

In summary, previous findings consistently indicate that there is an effect of SES on vocabulary: children from low SES score significant lower than their peers from mid-high SES. As for measures of verbal short-term memory with varied linguistic load, previous findings are inconclusive. There are studies showing that verbal short-term memory capacity, as a cognitive measure, is affected by SES (e.g., Jensen and Figueroa, 1975; Gardner et al., 2006), while other findings show no effect of SES for FWD (e.g., Engel et al., 2008). In a similar vein, findings provide inconclusive evidence for NWR. There is evidence that NWR is free of SES influence (e.g., Gardner et al., 2006; Balladares et al., 2016); however, other studies seem to show that NWR is affected by SES (e.g., Roy et al., 2014). The disparity in results has been linked to task differences. For example, some NWR tasks include pseudo-words that resemble the target language morphologically, while other tasks include nonwords that do not contain morphemes of the target language. Results for SRep are more consistent and point at group differences, corroborating the findings for vocabulary: children from low SES score significantly lower than children from mid-high SES (e.g., Gardner et al., 2006; Roy et al., 2014). Some studies demonstrate that the effect of SES on repetition tasks disappears once vocabulary is controlled for (e.g., Balladares et al., 2016), suggesting that the effect of SES is mainly driven by smaller vocabularies. However, there is also some evidence that the effect of SES is not limited to the verbal domain and that it affects children’s executive function skills as well (e.g., Klenberg et al., 2001; Ardila et al., 2005). The disparity between low and high SES has been attributed to impoverished linguistic input, genetic factors and numerous environmental factors (e.g., Klenberg et al., 2001; Ardila et al., 2005; Hoff, 2006; Fernald et al., 2013). For example, living in unprivileged environments is associated with decreased levels of safety, higher noise levels, exposure to toxins, inadequate nutrition and medical care and higher levels of stress and instability.

### Effects of Bilingualism

Similar to the effect of SES, previous studies consistently report lower vocabulary scores for bilingual children as compared to monolingual peers when tested in only one of their languages (especially the SL) (for an overview see Haman et al., 2015 and studies cited there). For repetition tasks, previous studies investigating effects of bilingualism report conflicting findings (for a detailed overview see Meir, 2017). Some studies show that the capacity of verbal short-term memory as measured by FWD is not affected by bilingualism (e.g., Bialystok and Shapero, 2005; Messer et al., 2010; Engel de Abreu, 2011; Blom and Boerma, 2017), while other studies show that bilinguals demonstrate limitation in verbal short-term storage in the SL (e.g., Laloi, 2015). Similar to the findings on FWD, many studies show that bilinguals perform on a par with monolinguals on NWR (e.g., Thordardottir and Brandeker, 2013). However, there are also some studies that demonstrate a negative effect of bilingualism (e.g., Messer et al., 2010; Engel de Abreu et al., 2013; Laloi, 2015). The differences in results across different language groups on NWR have been attributed to the stimulus type used.

Boerma et al. (2015), for example, looked at the effects of bilingualism on two types of Dutch NWR tasks (quasi-universal and language-specific) in children with and without SLI. A negative effect of bilingualism is reported only on the language-specific test, while on the quasi-universal test, which was designed to be minimally influenced by the knowledge of any specific language, there is no effect of bilingualism. A recent study by Chiat and Polišenská (2016) reports no effect of bilingualism for three types of English NWR tasks (quasi-universal with and without the prosody of the English language; language-specific). That is, children are better in repeating word-like nonwords (i.e., nonwords that contain real morphemes of the language, have higher phonotactic probability, and/or fall into dense lexical neighborhoods) (for an overview see Chiat, 2015). This is consistent with the claim that NWR is associated with vocabulary size (Gathercole, 2006) and might explain the gap between monolingual and bilingual children who might have smaller vocabulary in their SL. However, somewhat different findings are reported in the study by Messer et al. (2010), who compared the repetition of nonwords with high and low phonotactic probability in the target language in bilingual Turkish–Dutch and monolingual Dutch children. In Dutch (the SL for the bilingual children), the Turkish–Dutch children had more difficulty in repetition of nonwords with low phonotactic probability as compared to the Dutch monolingual children. The authors suggested that language input provided a possible explanation for the unexpected lower performance of bilingual children on the Dutch stimuli with low phonotactic probability. That is, in the case of monolingual children, a more extensive and longer period of input supported the storage of even relatively infrequent phoneme clusters in Dutch, an advantage that was not available for the Turkish–Dutch children for whom Dutch is the SL.

The findings for SRep, the measure with the highest linguistic load, also provide inconclusive evidence. Two groups of children (Russian–Hebrew and English–Hebrew) reported in Chiat et al. (2013) show performance comparable to monolingual peers, while in the other two groups (Turkish–English and Russian–German), a large portion of children score at risk for SLI. The difference there was associated with the lexical requirements of the SRep tasks used, which were more demanding for the latter groups, as well as the possible difference in SES between the different cohorts as the latter included more children from lower SES. That is, similarly to the results for the effects of SES on repetition tasks, differences between bilingual and monolingual children seem to be driven by vocabulary differences. For example, Engel de Abreu et al. (2013) showed that group differences disappeared once vocabulary size was taken into consideration. In the same vein, Komeili and Marshall (2013) also showed that monolingual-bilingual group differences on SRep disappeared when receptive vocabulary was controlled for.

To summarize, previous research shows that bilingualism is associated with decreased vocabulary size in the SL, whereas the results are inconclusive for repetition tasks with low and high linguistic load. While some studies report no bilingual effect, other studies demonstrate a negative effect of bilingualism. Yet the negative effect of bilingualism on repetition tasks has been linked to decreased vocabulary size and/or limited exposure.

### Independent and Combined Effects of SES and Bilingualism

There are very few studies that address effects of SES in bilingual children. To the best of our knowledge only three studies have attempted to evaluate the effects of SES and bilingualism. Calvo and Bialystok (2014) assessed the separate and combined effects of SES and bilingualism on receptive vocabulary, non-verbal intelligence and executive function tasks. Likewise, Chiat and Polišenská (2016) assessed the independent and combined effect of SES and bilingualism on receptive vocabulary, but also on NWR among English speaking children residing in the United Kingdom. Finally, Gathercole et al. (2016) evaluated the contribution to SES on vocabulary, grammar and cognitive skills in monolingual English, and English–Welsh bilinguals.

Each of these studies used different measures to determine the SES of the bilingual children, who had varied linguistic backgrounds. Calvo and Bialystok (2014) assessed four groups of children aged 6–7 years old residing in Canada: monolingual English speaking from working-class and middle-class families; and bilingual children from working-class and middle-class families. The bilingual children spoke 26 different languages. SES status was determined by mother’s years of education. Gathercole et al. (2016) also used parent’s educational level (five-point scale: 1 = primary education and five-post-graduate education) as well as parents’ occupation (four-point scale: 1 = elementary trades and services; 4 = corporate directors, health and science professionals). Similarly to the other two studies, one of the languages of the children was English, but the other language of the bilinguals was constant, Welsh. Some bilinguals had only English at home, others Welsh and English at home, and another group only Welsh at home. Finally, for Chiat and Polišenská (2016), children’s SES status was determined by neighborhood status (mid-high SES: inner-London neighborhood; low SES: outer-London neighborhood), rather than parental education. However, as in the study by Calvo and Bialystok (2014), bilingual children were of mixed ethnic and cultural origins. For example, bilingual children from mid-high SES were Spanish–English, while bilingual children from low SES were predominantly Turkish–English. Moreover, the groups were not matched for age, and age was included as a covariate.

As for cognitive abilities, neither SES, nor bilingualism had an effect on non-verbal intelligence in Calvo and Bialystok (2014). On executive function tasks, there was a negative influence of SES with working class children performing lower than middle-class children, while the effect of bilingualism was positive (bilingual children obtained higher scores than monolingual children). No interaction between SES and bilingualism was observed for any of these cognitive measures. In the same vein, Gathercole et al. (2016) report significant correlations between a composite SES score and cognitive measures. The authors also assess the contribution of the home language and SES for cognitive measures, and found that home language played no role, but SES significantly contributed to performance on cognitive tasks at ages 3 and 5.

Where vocabulary is concerned, the findings are rather systematic. Calvo and Bialystok’s (2014) findings revealed an effect of SES and an effect of bilingualism for receptive vocabulary. Children from working class families had smaller vocabularies than children from middle-class families. Similarly, bilingual children were found to have smaller vocabularies than monolingual children. No interaction between SES and bilingualism was found for receptive vocabulary, suggesting that SES affects monolingual and bilingual children’s vocabulary similarly. The results for receptive vocabulary in Chiat and Polišenská (2016) were in line with those reported for receptive vocabulary by Calvo and Bialystok (2014): there were significant main effects of SES and bilingualism, with no interaction between SES and bilingualism. The low SES groups showed lower vocabulary scores compared to mid-high SES groups, and bilingual children scored lower than monolingual children. The lack of interaction between SES and bilingualism indicates that SES affected monolingual and bilingual children similarly. Finally, Gathercole et al. (2016) also report significant correlations between a composite SES score and children’s performance on language measures (receptive vocabulary in English and Welsh; receptive grammar skills in English and Welsh) with higher influence of SES on language measures as compared to cognitive measures. The effect of home language is reported to be more influential at younger ages, while the influence of SES is observed to be more influential at later ages.

Only Chiat and Polišenská (2016) explored the effects of SES and bilingualism on NWR tasks using three tasks that vary in their use of knowledge stored in long-term verbal memory: cross-linguistic (compatible with different languages and prosody neutral); prosodic specific (the same items as in cross-linguistic but with English real-word-like prosody) and language-specific (containing features specific to the target language, English, e.g., word-like derivational morphemes). All three repetition tasks were found to be free of SES and bilingualism effects. The authors indicated that the effect of SES was approaching significance for a language-specific NWR task. Finally, when vocabulary size was controlled for, these non-significant differences disappeared.

To recap, the three studies assessing independent effects of SES and bilingualism converge in showing that SES affects monolingual and bilingual children similarly. However, the factorial design used in the study by Calvo and Bialystok (2014) pointed out that SES and bilingualism affect different domains. As for the effect of SES, it was shown to affect both domains (language skills and cognitive skills). Conversely, bilingualism is associated with a lower performance on language tasks only, while it demonstrates an increase on the executive function tasks. Similarly, the factorial design applied to the three NWR tasks in the study by Chiat and Polišenská (2016) also demonstrate that SES is associated with the linguistic load that the tasks carries. A negative effect of SES appears only on the NWR task with greater linguistic load (i.e., on the language specific task). While two of these studies were the first to attempt to evaluate independent and combined effects of SES and bilingualism, the mixed ethnicity and cultural background might have affected the overall results. Previous studies point out the potential effects of cultural and racial difference which were not addressed (e.g., Calvo and Bialystok, 2014; Chiat and Polišenská, 2016). Like Gathercole et al. (2016), the present study controls for this confound by looking at bilingual children who share the same cultural background and share the same home language (here HL-Russian). Following the above studies, the present paper evaluates independent and combined effects of SES and bilingualism on vocabulary, but adds three measures of verbal short-term memory with varying linguistic load (FWD, NWR, and SRep). Secondly, this paper aims to investigate the relation between vocabulary and verbal short-term memory.

## Materials and Methods

### Participants

A total of 120^1^ monolingual Hebrew-speaking and sequential Russian–Hebrew speaking children with typical language development, aged 5; 7–6; 7, participated in the current study. The children were split into four groups: bilingual children with low SES (bi-LOW: *n* = 44) and mid-high SES (bi-MID-HIGH: *n* = 44); monolingual children with low SES (mo-LOW: *n* = 16) and mid-high SES (mo-MID-HIGH: *n* = 16). See **Table 1** for the information on the participants. Bilingual children and monolingual Hebrew-speaking children were living in the central part of Israel (Tel-Aviv area). Background information was collected from parents on family history as well as aspects of language development and developmental milestones using a short version of the bilingual parental questionnaires (BIPAQs) (Abutbul-Oz et al., 2012). The four groups were matched for age [*F*(3,116) = 0.85, *p* = 0.47] and non-verbal IQ as measured by the *Raven’s Colored Progressive Matrices Non-verbal IQ Test* (Raven, 1998) [*F*(3,116) = 2.10, *p* = 0.10].

**Table 1:** Background information [Means (SDs) and Ranges] on the participants per group.

	Bilingual		Monolingual	
	bi-LOW	bi-MID-HIGH	mo-LOW	mo-MID-HIGH
	*N* = 44	*N* = 44	*N* = 16	*N* = 16
Age in months	73 (3) 67–79	73 (2) 68–77	72 (2) 68–77	73 (1) 71–75
Non-verbal IQ (raw score out of 36)	20 (4) 12–33	22 (4) 13–30	19 (2) 16–24	20 (4) 13–27
Mother’s education (years)	11 (1) 10–12	16 (2) 13–25	12 (1) 10–12	16 (1) 14–19
Fathers’ education (years)^a^	12 (2) 10–17	15 (3) 8–25	12 (1) 10–15	17 (4) 12–23
AoO (age of onset of bilingualism) in months	33 (22) 0–60	32 (21) 0–60	n/a	n/a
LoE (length of exposure to L2) in months	39 (21) 13–74	41 (21) 13–76	n/a	n/a

*^a^Data on father’s education were missing for 13 participants (six bi-LOW, four bi-HIGH, two mo-LOW, one bi-HIGH).*

*bi-LOW: bilingual children from low SES; bi-MID-HIGH: bilingual children from mid-high SES; mo-LOW: monolingual children from low SES; mo-MID-HIGH: monolingual children from mid-high SES.*

By definition, the groups differed on the SES parameter, which was operationalized by years of maternal education [*F*(3,116) = 80.76, *p* < 0.001, $\eta_{p}^{2}$= 0.68], and *post hoc* tests using Tamhane’s T2 for unequal variances confirmed SES differences: (mo-LOW = bi-LOW) < (mo-MID-HIGH = bi-MID-HIGH). Similarly, there were group differences for father’s years of education [*F*(3,103) = 19.11, *p* < 0.001, $\eta_{p}^{2}$= 0.36], and *post hoc* tests using Tamhane’s T2 for unequal variances confirmed SES differences: (mo-LOW = bi-LOW) < (mo-MID-HIGH = bi-MID_HIGH). There were significant correlations between father and mother’s education (*r* = 0.67, *n* = 107, *p* < 0.001).

The two bilingual groups were matched by the age of Hebrew onset [*F*(1,86) = 0.16, *p* = 0.69] and the length of exposure to Hebrew [*F*(1,86) = 0.25, *p* = 0.62]. We also measured bilingual children’s expressive vocabulary in HL-Russian using naming subtests of the *Russian Language Proficiency Test for Multilingual Children* (Gagarina et al., 2010), which includes naming of nouns and verbs. The results using an independent *t*-test indicated that the two bilingual groups (bi-LOW and bi-MID-HIGH) did not differ in their vocabulary size in HL-Russian [*t*(86) = -1.86, *p* = 0.32].

### Tasks

Children were tested with a battery of tasks to explore language proficiency in Hebrew, language proficiency in Russian (for bilingual children only), and three repetition tasks in SL-Hebrew (FWD, NWR, and SRep).

• **Expressive Vocabulary in Hebrew:** The naming subtest of the *Goralnik Screening Test for Hebrew* (Goralnik, 1995) was used as a measure of children’s expressive vocabulary in Hebrew.
• **Hebrew Forward Digit Span (FWD)**: The Hebrew FWD Task, adapted from Wechsler Intelligence Scale for Children (WISC-R95), was administered to all children.
• **Hebrew Nonword repetition (NWR):** Shortened version of the Hebrew NWR task (Armon-Lotem and Chiat, 2012), which is comprised of 14 items was administered. The nonwords were constructed to include the following variables: item length (2–4 syllabic items); consonant sequences (with or without a consonant sequence); word-likeness (word-like vs. nonword-like). All nonwords were constructed using non-existent roots. Half of the nonwords made use of typical consonant and vowel patterns for Hebrew (word-like nonwords), and half of the words made use of vowelled templates that are atypical of Hebrew.
• **Hebrew Sentence Repetition (SRep):** The Hebrew LITMUS-SRep task (Meir et al., 2016), which includes 56 sentences, was administered. LITMUS-SRep tasks followed the guidelines developed within COST Action IS0804 (Marinis and Armon-Lotem, 2015).

### Procedure and Coding

Informed parental consent was obtained prior to participation for each child. The study was approved by the review board of Bar-Ilan University as well as by the Israeli Ministry of Education. Each participant was tested individually in a quiet room in the preschool or at home. This study is part of a larger study in which bilingual participants were tested in both languages (Russian and Hebrew). The tasks were administered in two sessions: (1) the language proficiency test in Hebrew (which includes a vocabulary subtest), a FWD and the *Raven’s Colored Progressive Matrices Non-verbal IQ Test* (Raven, 1998); (2) NWR and SRep tasks. All children in the current study completed all the tasks.

The FWD, NWR, and SRep tasks were pre-recorded for consistency of presentation. The tasks were presented via a power-point presentation, earphones, and a microphone. The child heard each stimulus only once, and was instructed to repeat it verbatim. Children’s responses were recorded using Audacity software^2^ and were marked as correct/incorrect on-line. Recordings were then transcribed and re-coded off-line.

#### Expressive Vocabulary

The children were presented with 15 objects with different levels of familiarity and were asked to name them. The coding system in the current study was different from the original coding schema used in the *Goralnik Screening Test for Hebrew* (Goralnik, 1995). For consistency of presentation of all tasks, raw scores were converted into a ratio out of the15 items presented.

#### Forward Digit Span

The children were asked to repeat the digit sequence orally. Test items consisted of two lists of digits administered for each list length, beginning with a length of two digits, and increasing in length by one digit following successful repetition of at least one list of digits at a given length. The test was discontinued when the child failed at two consecutive digit sequences of the same length. The longest list length correctly repeated was noted.

#### Nonword Repetition

The children’s repetitions of the nonwords were scored as correct (a score of 1) if all consonant and vowels were produced correctly. If the response contained any substitution, omission, or insertion, it was scored as incorrect and given a score of 0. Raw scores were converted to a proportion out of the 14 items tested. All children were able to complete the entire task.

#### Sentence Repetition

A 0–1 scoring scheme was used for SRep, according to which a score of 1 was allocated if the sentence was repeated entirely verbatim and a score of 0 if there were one or more changes in the child’s response. Raw scores were converted to a proportion out of the 56 items targeted. The entire task was presented to all participants.

### Analysis

To evaluate independent effects of SES and bilingualism, two-way ANOVAs with SES (low SES vs. mid-high SES) and language group (monolingual vs. bilingual) as independent factors were applied. Combined effects of SES and language group were determined by interactions between SES and bilingualism. To estimate the magnitude of each factor, effect sizes were determined by partial eta squared (partial η^2^). In order to evaluate the effect of vocabulary on the relationship between SES and repetition tasks, following Komeili and Marshall (2013) and Balladares et al. (2016), we additionally conducted two analyses of variance using expressive vocabulary and FWD as covariates. Finally, outliers (participants performing below -2 SD and above 2 SD) for each group on each measure were identified. Yet excluding these outliers did not affect the statistics reported in Section “Results.”

## Results

### The Effect of SES and Bilingualism on Vocabulary in Hebrew

**FIGURE 1:**
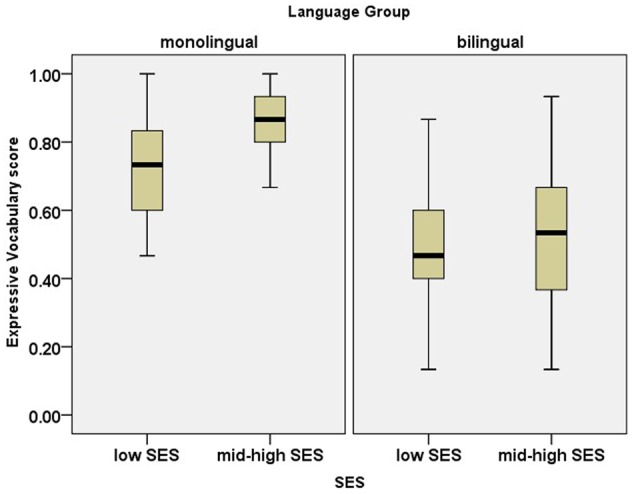
Box plots for scores on the expressive vocabulary task. The plots show the median (thick line within box), 25th and 75th percentiles (box), 10th and 90th percentiles (whiskers), outliers (circles) and extreme outliers (stars).

To examine the effect of SES and bilingualism on vocabulary in Hebrew (SL for bilingual children), a two-way ANOVA with SES (low vs. mid-high) and language group (monolingual vs. bilingual) was conducted. **Figure 1** presents children’s vocabulary scores. The results indicated a significant effect of SES [*F*(1,116) = 5.88, *p* = 0.02, $\eta_{p}^{2}$= 0.05], a significant effect of language group [*F*(1,116) = 48.53, *p* < 0.001, $\eta_{p}^{2}$= 0.29] and no interaction between SES and language group [*F*(1,116) = 1.35, *p* = 0.25]. The analysis indicated that the effect size of language group for vocabulary size is higher than the effect size of SES (compare $\eta_{p}^{2}$^.^29 vs. 0.05).

### The Effect of SES and Bilingualism on Tasks Tapping into Verbal STM (FWD, NWR, and SRep) in Hebrew

The effects of SES and bilingualism were further explored for the different repetition tasks that tap into verbal STM but vary in linguistic load. Two-way ANOVAs SES (low vs. mid-high) and language group (monolingual vs. bilingual) were conducted for the three repetition tasks (FWD, NWR, and SRep) in Hebrew (the SL for the bilingual children).

#### Forward Digit Span (FWD) Task

**FIGURE 2:**
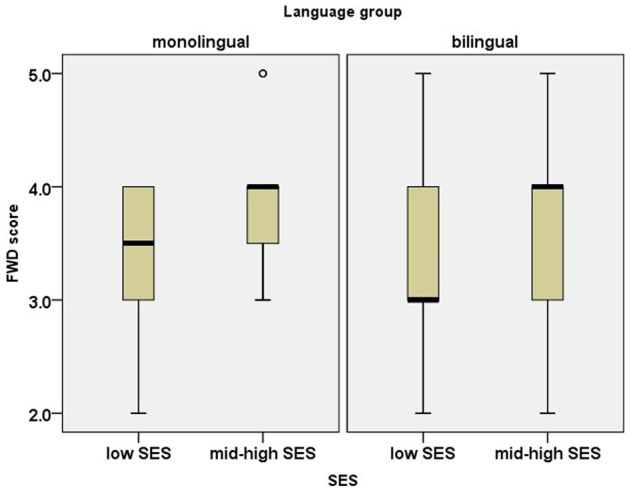
Box plots for scores on the forward digit span (FWD) task. The plots show the median (thick line within box), 25th and 75th percentiles (box), 10th and 90th percentiles (whiskers), outliers (circles), and extreme outliers (stars).

**Figure 2** depicts children’s performance on the Hebrew FWD task. A two-way ANOVA with SES (low vs. mid-high) and language group (monolingual vs. bilingual) as independent variables indicated a significant effect of SES [*F*(1,116) = 11.17, *p* < 0.001, $\eta_{p}^{2}$= 0.09], but no effect of language group [*F*(1,116) = 0.29, *p* = 0.59], and no interaction between language group and SES [*F*(1,116) = 0.38, *p* = 0.54].

#### Nonword Repetition (NWR) Task

**FIGURE 3:**
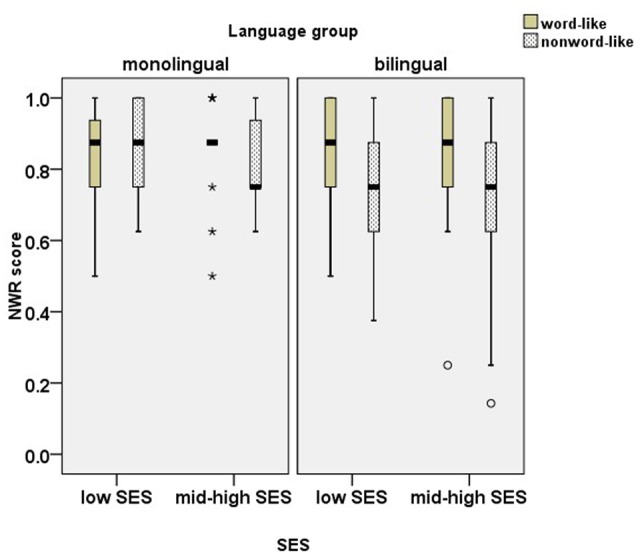
Box plots for scores on the nonword repetition (NWR) task (comparison of word-like vs. nonword-like items). (a) The plots show the median (thick line within box), 25th and 75th percentiles (box), 10th and 90th percentiles (whiskers), outliers (circles), and extreme outliers (stars). (b) In the monolingual mid-high SES group, there were 10 participants, whose score was 0.88, and 6 extreme outliers (three participants with the scores of 1.00), represented by a star above the thick line, and three participants with low scores, represented by three stars below the thick line.

The results for the total score for the Hebrew NWR task failed to show a significant effect of either bilingualism [*F*(1,116) = 1.79, *p* = 0.18]; or SES [*F*(1,116) = 0.05, *p* = 0.82], or interaction between SES and bilingualism [*F*(1,116) = 1.79, *p* = 0.18]. Following previous observation that NWR accuracy depends on the type of stimuli, we conducted a further analysis assessing the nonword type stimuli. **Figure 3** presents the scores on NWR by stimulus type.

A three-way ANOVA with stimulus type (word-like vs. nonword-like) as a within-subject factor and SES (low vs. mid-high) and language group (monolingual vs. bilingual) as between-subject factors revealed that the effect of stimulus type was marginally significant [*F*(1,112) = 3.71, *p* = 0.06, $\eta_{p}^{2}$= 0.09]. There was no significant main effect of SES [*F*(1,112) = 2.08, *p* = 0.15], no significant main effect of language group [*F*(1,112) = 2.59, *p* = 0.11] and no interaction between SES and language group [*F*(1,116) = 0.47, *p* = 0.50]. There was no stimulus type and SES interaction [*F*(1,112) = 0.30, *p* = 0.59]; however, there was a significant stimulus type by language group interaction [*F*(1,112) = 7.14, *p* = 0.01, $\eta_{p}^{2}$= 0.06]. In order to unpack the interactions, separate one-way ANOVAs were conducted for word-like and nonword-like stimuli with language group (monolingual vs. bilingual) as an independent variable. The results showed no effect of language group for word-like stimuli [*F*(1,114) = 0.00, *p* = 0.99]; however, a negative effect of bilingualism was observed for nonword-like stimuli [*F*(1,114) = 7.39, *p* = 0.01, $\eta_{p}^{2}$= 0.06].

#### Sentence Repetition (SRep) Task

**FIGURE 4:**
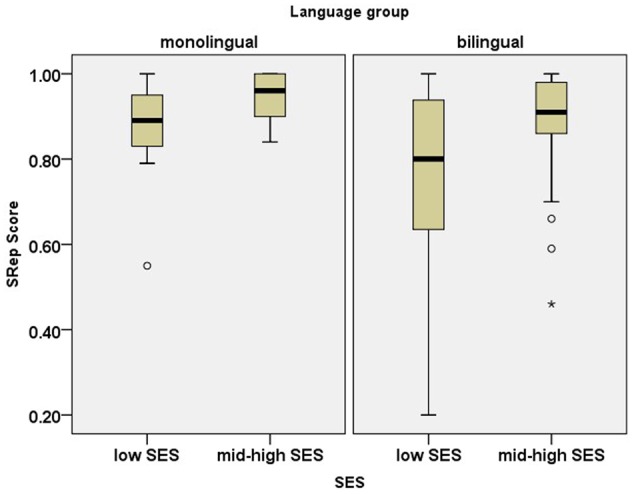
Box plots for scores on the Hebrew Sentence Repetition (SRep) task. The plots show the median (thick line within box), 25th and 75th percentiles (box), 10th and 90th percentiles (whiskers), and outliers (circles), and extreme outliers (stars).

**Figure 4** presents children’s scores on the Hebrew SRep Task. The analysis showed a significant effect of SES [*F*(1,116) = 8.56, *p* < 0.001, $\eta_{p}^{2}$= 0.07], and a significant effect of language group [*F*(1,116) = 14.55, *p* < 0.001, $\eta_{p}^{2}$= 0.05] but no interaction between SES and language group [*F*(1,116) = 0.16, *p* = 0.69]. The effect size of SES and the effect of bilingualism were comparable (compare $\eta_{p}^{2}$: 0.07 vs. 0.05).

### The Relationship between Vocabulary, SES, and Bilingualism

To assess the role of vocabulary size in the impact of SES and bilingualism on the repetition tasks, we re-analyzed the performance on FWD, NWR and SRep using a two-way ANCOVA with SES (low vs. mid-high) and language group (monolingual vs. bilingual) as independent variables and the expressive vocabulary scores as a covariate.

The analysis of FWD showed that the effect of SES persisted even after the vocabulary size was taken into consideration [*F*(1,115) = 7.92, *p* = 0.01, $\eta_{p}^{2}$= 0.06], while the effect of language group [*F*(1,115) = 0.62, *p* = 0.43] and the SES^∗^language group interaction [*F*(1,116) = 0.15, *p* = 0.70] remained insignificant.

The re-analysis of NWR, using a three-way ANCOVA with stimulus type (word-like vs. nonword-like) as a within-subject factor and SES (low vs. mid-high) and language group (monolingual vs. bilingual) as between-subject factors, with expressive vocabulary scores as a covariate, showed that all the main effects remained non-significant [stimulus type: *F*(1,111) = 1.27, *p* = 0.26; SES: *F*(1,111) = 1.12, *p* = 0.29; language group: *F*(1,111) = 0.01, *p* = 0.91; SES^∗^language group : *F*(1,111) = 2.34, *p* = 0.13] and the stimulus type^∗^ language group interaction became non-significant as well [*F*(1,111) = 3.61, *p* = 0.06]. The effect of bilingualism, which was observed on nonword-like stimuli, was largely driven by vocabulary knowledge, and it disappeared once vocabulary size in Hebrew (the SL for bilinguals) was taken into account.

Likewise, the analysis for the SRep task indicated that the observed effects of language group disappeared once vocabulary size was controlled for [language group: *F*(1,110) = 0.19, *p* = 0.67; SES^∗^language group : *F*(1,110) = 0.59, *p* = 0.45], while the effect of SES persisted [*F*(1,110) = 5.18, *p* = 0.02, $\eta_{p}^{2}$= 0.04]. Subsequently, we re-analyzed the data for the SRep task, using forward digit scores and vocabulary scores as covariates. The results indicated that once FWD scores were added as a covariate, the effect of SES disappeared [SES: *F*(1,108) = 2.40, *p* = 0.12].

To summarize this subsection, these findings demonstrated independent effects of SES and bilingualism. The negative effect of bilingualism on tasks with higher linguistic load (nonword-like items and SRep) was associated with smaller vocabulary sizes in bilingual children in their SL. Yet the negative effect of SES was not linked to vocabulary size. That is, it did not disappear on the FWD task and the SRep task when vocabulary was controlled for. Moreover, the negative effect of SES on SRep was found to be related to the forward digit task that measures memory and relies the least on long-term verbal memory.

## Discussion

Previous studies consistently demonstrated a negative effect of SES on language development that often leads to variation within the pathological range, but previous research was less consistent for the effects of bilingualism. Few studies explored the interaction between these two variables, looking at independent and combined effects. Moreover, these studies have only enhanced the inconsistency, possibly due to linguistic and cultural heterogeneity among the bilingual children, and wide age-range involved. Furthermore, in most of the previous work, children were tested in English as the SL. The present study controlled for the linguistic background of the children, as all bilinguals had the same HL, Russian, and both bilinguals and monolinguals came from the same neighborhoods and spoke the same SL, Hebrew. Children were matched for age and IQ, and SES was consistently determined by maternal education. All children were tested with the same tools looking at expressive vocabulary and three repetition tasks (FWD, NWR, and SRep) tapping into verbal short-term memory.

Regarding vocabulary size, the results of our meticulous approach were consistent with previous work, showing that both SES and bilingualism have an impact. In line with previous research, monolingual Hebrew speaking children outperformed Russian–Hebrew bilinguals in the SL (Hebrew). Furthermore, children of mid-high SES outperformed those of low SES.

As for the independent effect of SES, the results of the present study indicated that children from lower SES backgrounds score lower on FWD and SRep. FWD measures verbal short-term memory as a cognitive capacity and is less associated with vocabulary, while SRep task is the measure with the highest linguistic load, tapping into long-term linguistic representations. Interestingly, no effect of SES was found for the NWR task, neither for word-like, nor for nonword-like stimuli. The present results are consistent with studies suggesting that NWR is free of SES influence (e.g., Gardner et al., 2006; Balladares et al., 2016; Chiat and Polišenská, 2016). However, the discussion is still open regarding the exact mechanisms involved in NWR. The question is why NWR, which taps into verbal short-term memory capacity, on the one hand, and vocabulary, on the other hand, remains free of SES influence, whereas both verbal short-term memory and vocabulary are negatively affected by low SES. A major difference between NWR and FWD (and to some extent SRep as well) is that NWR does not measure memory span, but rather phonological processing. In NWR, only a single word is held in memory at each point. This might suggest that the difficulty with FWD stems from the need to hold several items in short-term memory rather than the linguistic challenge presented by NWR. This distinction further supports our conclusion that SES interferes with cognitive abilities.

Turning to the independent effect of bilingualism on repetition tasks, previous research brought conflicting evidence. The present study shows that the effect of bilingualism varied on repetition tasks as a function of linguistic load. No effect of bilingualism was detected for verbal short-term capacity as measured by FWD. However, as the linguistic load rises on repetition tasks, so does the effect of bilingualism. This replicates the findings by Calvo and Bialystok (2014), who reported the negative effect of bilingualism only for linguistic tasks. Moreover, a negative effect of bilingualism was observed on the NWR task only for nonword-like items (i.e., items which carry no morpho-lexical information in Hebrew, the SL for bilingual children). These results were surprising, as previous studies showed that bilinguals perform similarly to monolinguals on quasi-universal non-repetition tasks (tasks designed to minimize the influence of knowledge and exposure to any particular language (e.g., Boerma et al., 2015; Chiat and Polišenská, 2016). The discrepancy in results might be attributed to nonword properties. Quasi-universal nonwords are constructed from a limited range of consonants and vowels which are combined into simple CVCV structures. Only those consonants and vowels that are compatible with word phonology in most languages, regardless of the further segmental contrasts and syllable structures particular languages allow, were chosen. In contrast, in the present NWR task, nonword-like items were designed in a different way, using almost the full range of Hebrew consonants and vowels in non-Hebrew-like vowelled templates, and this resulted in differences between monolingual and bilingual children. The results of the present study echo those reported in Messer et al. (2010), who found that in Dutch (the SL for the bilingual children), the Turkish–Dutch children had more difficulty with repetition of nonwords with low phonotactic probability as compared to their monolingual Dutch peers. One of the explanations for lower performance in bilinguals on nonwords with low phonotactic probability in their SL might be the reduced language input in the SL. More extensive exposure in monolingual children enables the storage of infrequent phoneme clusters, while for bilingual children this advantage was not yet available (Messer et al., 2010). An alternative explanation might relate to bilingual processing, which activates both systems before focusing the attention on the relevant system. In repeating nonwords that are similar to the SL, bilingual children, like monolinguals, are quickly able to identify the SL morpho-lexical information that facilitates their repetition. But once the nonwords are no longer similar to one of the systems, bilinguals might find it more difficult to resolve the competition, thus challenging verbal short-term memory. This option is not available to monolingual children. Neither is it relevant for quasi-universal nonwords as they are compatible with phonotactic rules of both languages, and quasi-universal nonwords gain support from mental lexicons of both languages.

Besides evaluating independent effects of SES and bilingualism, we assessed the combined effects of these two variables on vocabulary and repetition tasks. The results for the combined effects conformed to previous research showing no interaction between SES and bilingualism (e.g., Calvo and Bialystok, 2014; Chiat and Polišenská, 2016). These findings suggest that SES similarly affects bilingual and monolingual children, and that bilingualism affects similarly children from low and mid-high SES. However, similarly to Calvo and Bialystok (2014), our study shows that SES and bilingualism impact different domains. We found that both SES and bilingualism affect vocabulary and repetition tasks with the highest linguistic load (e.g., SRep). However, the capacity of verbal short-term memory (a cognitive measure less associated with vocabulary) is affected by SES, but not by bilingualism.

This was confirmed by exploring the relationship between vocabulary size, SES and bilingualism. The present study shows, as found in previous studies, that the negative influence of bilingualism is largely driven by the smaller vocabulary size of bilingual children in the SL (here Hebrew). Once vocabulary size is accounted for, the negative effect of bilingualism disappeared, pointing to the fact that the bilingual children’s lower performance in the SL demonstrates that they are disadvantaged due to lesser experience in that language. Conversely, the negative effect of low SES persisted on the FWD and SRep tasks when vocabulary scores were taken into consideration. The negative effect of SES disappeared when the measure of verbal short-term memory with lowest linguistic load (FWD) was added to the model. Indeed, previous research demonstrated that a negative effect of SES is not limited to the verbal domain. Living in underprivileged backgrounds which provide less adequate social and cognitive stimulation affects children’s language and cognitive abilities (e.g., Klenberg et al., 2001; Bradley and Corwyn, 2002; Ardila et al., 2005; Hoff, 2006; Fernald et al., 2013). These differences between low and high SES have been attributed to genetic factors (see Bradley and Corwyn, 2002; Fernald et al., 2013). However, a study on twins (Turkheimer et al., 2003) showed that in impoverished families 60% of the variance in children’s IQ is accounted for by the shared environment, and the contribution of genes is close to zero. These latter findings provide hope that children’s lower verbal and cognitive skills can be improved if educational settings accommodate needs of children from low SES for more stimulating environments.

To conclude, the present study is the first to assess independent and combined effects of SES and bilingualism on expressive vocabulary and three repetition tasks (FWD, NWR, and SRep) which tap into verbal short-term memory. It provides new evidence for the distributed impact of SES and bilingualism on the development of preschool children as it has shown that SES and bilingualism impact different abilities of children, yielding variation in their linguistic and cognitive profiles.

Bilingualism is associated with decreased vocabulary size and lower performance on verbal short-term memory tasks with higher linguistic load in the SL. The negative effect of bilingualism on verbal short-term memory evaporates once vocabulary is accounted for. That is, our study shows that bilingualism impacts language development at the lexical level as child vocabulary in the SL is more restricted when compared to that of monolingual. We did not find a cumulative negative bilingual effect on tasks which rely on long and short-term memory, as the difference between bilinguals and monolinguals disappears once lexical abilities in the SL are controlled for. These findings could be interpreted as suggesting that bilinguals’ representation of the SL is similar to that of monolinguals, while errors made by bilinguals are related to bilingual processing and gaps in lexical knowledge. Our findings, while not showing a cognitive advantage for bilingual children, do not show a disadvantage either.

Turning to SES the story is very different. SES influences not only linguistic performance, but also verbal short-term memory with lowest linguistic load. The negative effect of SES cannot be attributed solely to lower vocabulary scores, suggesting that an unprivileged background has a negative impact on children’s cognitive development beyond a linguistic disadvantage. That is, while bilingualism impacts lexical knowledge only, our findings show a cumulative effect of lexical knowledge and memory-related cognitive skills on the performance of children of low SES. These findings suggest that SES has a negative impact on short and long-term memory. That is, cognitive abilities are tied to socio-genetic factors associated with low SES. The results of the current study have important clinical implication, indicating that caution should be employed when assessing the language and cognitive development of children from diverse communities.

## Ethics Statement

This study was carried out in accordance with the recommendations of Bar Ilan University review board for studies involving human subjects as well as by the Israeli Ministry of Education with written informed consent from all parents and the approval of all subjects. All parents gave written informed consent in accordance with the Declaration of Helsinki. The protocol was approved by Bar Ilan University review board for studies involving human subjects as well as by the Israeli Ministry of Education.

## Author Contributions

NM and SA-L both developed, conceptualized the research questions and the design of the study, and wrote the manuscript. NM carried out the data analyses.

## Conflict of Interest Statement

The authors declare that the research was conducted in the absence of any commercial or financial relationships that could be construed as a potential conflict of interest.

## Footnotes

1 Children reported in this study were drawn from a larger pool of 230 participants (see Armon-Lotem and Meir, 2016). In the current study, we included only children with typical language development.

2 www.audacity.com
